# Integrating MALDI-MSI-Based Spatial Proteomics and Machine Learning to Predict Chemoradiotherapy Outcomes in Head and Neck Cancer

**DOI:** 10.3390/ijms26189084

**Published:** 2025-09-18

**Authors:** Marta Grzeski, Patrick Moeller Jensen, Benjamin-Florian Hempel, Herbert Thiele, Jan Lellmann, Simon Schallenberg, Volker Budach, Ulrich Keilholz, Ingeborg Tinhofer, Oliver Klein

**Affiliations:** 1 Imaging Mass Spectrometry Unit, Berlin Institute of Health, Charité – Universitätsmedizin Berlin, 13353 Berlin, Germany; marta.grzeski@charite.de (M.G.); benjamin.hempel@fu-berlin.de (B.-F.H.); 2 Department of Applied Mathematics and Computer Science, Technical University of Denmark, 2800 Kongens Lyngby, Denmark; patmjen@dtu.dk; 3 Veterinary Center for Resistance Research, Department of Veterinary Medicine, Freie Universität Berlin, 14163 Berlin, Germany; 4 Fraunhofer Institute for Digital Medicine MEVIS, 23562 Lübeck, Germany; herbert.thiele@mevis.fraunhofer.de; 5 Institute of Mathematics and Image Computing, University of Lübeck, 23562 Lübeck, Germany; jan.lellmann@uni-luebeck.de; 6 Institute of Pathology, Charité – Universitätsmedizin Berlin, 10117 Berlin, Germany; simon.schallenberg@charite.de; 7 Department of Radiooncology and Radiotherapy, Charité – Universitätsmedizin Berlin, 13353 Berlin, Germany; prof.budach@radiotherapy.care (V.B.); ingeborg.tinhofer@charite.de (I.T.); 9 German Cancer Consortium (DKTK), Partner Site Berlin, a Partnership Between DKFZ and Charité – Universitätsmedizin Berlin, 10115 Berlin, Germany; 8 Charité Comprehensive Cancer Center, Charité – Universitätsmedizin Berlin, 10117 Berlin, Germany; ulrich.keilholz@charite.de

**Keywords:** MALDI-MSI, spatial proteomics, machine learning, prognostic classifier, head and neck cancer, chemoradiotherapy outcome

## Abstract

Head and neck squamous cell carcinoma (HNSCC) is often diagnosed at advanced stages. Due to pronounced intratumoral heterogeneity (ITH), reliable risk stratification and prediction of treatment response remain challenging. This study aimed to identify peptide signatures in HNSCC tissue that are associated with treatment outcomes in HPV-negative, advanced-stage HNSCC patients undergoing 5-fluorouracil/platinum-based chemoradiotherapy (CDDP-CRT). We integrated matrix-assisted laser desorption/ionization mass spectrometry imaging (MALDI-MSI) of tryptic peptides with univariate statistics and machine learning approaches to uncover potential prognostic patterns. Formalin-fixed, paraffin-embedded whole tumor sections from 31 treatment-naive, HPV-negative HNSCC patients were digested in situ with trypsin, and the generated peptides were analyzed using MALDI-MSI. Clinical follow-up revealed recurrence or progression (RecPro) in 20 patients, while 11 patients showed no evidence of disease (NED). Classification models were developed based on the recorded peptide profiles using both unrestricted and feature-restricted approaches, employing either the full set of *m*/*z* features or a subset of the most discriminatory *m*/*z* features, respectively. The unrestricted model achieved a balanced accuracy of 71% at the patient level (75% sensitivity, 66% specificity), whereas the feature-restricted model reached a balanced accuracy of 72%, showing increased specificity (92%) but reduced sensitivity (52%) in the CDDP-CRT cohort. In order to assess treatment specificity, models trained on the CDDP-CRT cohort were tested on an independent patient cohort treated with mitomycin C-based CRT (MMC-CRT). Neither model demonstrated prognostic performance in the MMC-CRT patient cohort, suggesting specificity for platinum-based therapy. Presented findings highlight the potential of MALDI-MSI–based proteomic profiling to identify patients at elevated risk of recurrence following CDDP-CRT. This approach may support more personalized risk assessment and treatment planning, ultimately contributing to improved therapeutic outcomes in HPV-negative HNSCC.

## 1. Introduction

Head and neck squamous cell carcinoma (HNSCC) is the most common malignancy of the head and neck, arising from the mucosal epithelium of the oral cavity, pharynx, and larynx. HNSCC is the seventh most prevalent cancer globally, and its incidence continues to rise, with projections indicating an increase of 30% by 2030 [1,2,3]. The majority of HNSCC patients are diagnosed at advanced stages (III and IV) and require multimodal treatment strategies, such as surgery combined with (chemo)radiotherapy or definitive chemoradiotherapy (CRT). CRT remains the standard first-line treatment for managing unresectable, locally advanced HNSCC [4]. Randomized studies and a meta-analysis have demonstrated a survival benefit from adding chemotherapy to RT [5]. However, a significant proportion of HNSCC patients still experience cancer recurrence or progression, posing a major clinical challenge in the management of HNSCC [6]. Moreover, concurrent CRT is associated with substantial toxicity, resulting in long-term side effects that impair the quality of life in patients and increase the risk of non-cancer-related mortality [7]. Consequently, patient stratification prior to treatment initiation is critical to avoid unnecessary exposure to toxic therapies for those unlikely to benefit. 

Reliable biomarkers for HNSCC remain scarce, despite extensive research on prognostic and predictive markers [8]. In clinical practice, treatment decisions are primarily guided by clinicopathologic factors such as tumor location and the TNM staging system [9,10]. While these factors provide insights into tumor morphology, extent, and size, they offer limited prognostic value and fail to reliably predict treatment response, as evidenced by the diverse clinical outcomes observed in patients with the same disease stage [11].

Although substantial progress has been made in understanding the molecular pathways driving HNSCC, and numerous studies have proposed novel clinical factors and biomarkers, routine clinical practice still lacks reliable tools to inform treatment decisions based on a tumor’s individual genetic or biological profile [8,12,13]. Given the well-described complexity and pronounced intratumoral heterogeneity (ITH) of HNSCC, comprehensive molecular profiling of HNSCC lesions requires the application of untargeted, spatially resolved analytical techniques.

These requirements are effectively met by matrix-assisted laser desorption/ionization mass spectrometry imaging (MALDI-MSI), a technique that combines the high sensitivity of mass spectrometry with the spatial resolution typically associated with histological staining. Precisely, by acquiring separate mass spectra at each XY coordinate across the tissue, MALDI-MSI generates spatially resolved molecular maps that reflect the inherent biochemical composition of the tissue. Notably, unlike conventional histological assessments that rely on the prior selection of specific markers, MALDI-MSI enables spatially resolved, yet untargeted analysis of a wide range of analytes—including metabolites, proteins, peptides, lipids, and glycans—within a single tissue section [14,15,16]. MALDI-MSI has been successfully applied to tumor histotype classification [17,18], therapy stratification [19], and predicting disease outcome [20]. In HNSCC, MALDI-MSI has been used to distinguish morphologically distinct regions in tumor tissue and reveal intratumoral heterogeneity that may correlate with disease progression [21,22].

As a consequence of providing comprehensive and spatially resolved molecular signatures of tissue, MALDI-MSI generates large volumes of complex data, the analysis of which is both time- and computationally intensive. Recent advances in machine learning have enabled efficient dimensionality reduction in the high-dimensional datasets produced by MALDI-MSI, facilitating the identification of meaningful molecular signatures. By isolating relevant features, machine learning models can improve diagnostic accuracy, enable patient stratification, and offer deeper insights into molecular heterogeneity [17,23,24,25].

In this pilot study, we integrate MALDI-MSI with univariate statistical analyses and machine learning to identify tryptic peptide signatures associated with treatment outcomes in HNSCC patients subjected to 5-fluorouracil/platinum-based chemoradiotherapy (CDDP-CRT). Using pre-treatment FFPE tumor specimens from patients with locally advanced, HPV-negative HNSCC, we focus on a subgroup known for poor therapeutic response. By leveraging the spatial resolution of MALDI-MSI and advanced statistical modeling, we aim to assess the prognostic accuracy of in situ proteomic signatures that could inform personalized treatment strategies. This approach holds promise for identifying patients at higher risk of recurrence, thereby supporting more informed clinical decision-making and potentially improving therapeutic outcomes.

## 2. Results

In this study, we investigated whether MALDI-MSI of tryptic peptides in combination with machine learning could identify putative in situ proteomic signatures predictive of treatment outcome in patients with advanced-stage, HPV-negative HNSCC treated with concurrent 5-fluorouracil (5-FU) and cisplatin-based chemoradiotherapy (CDDP-CRT). The study material consisted of FFPE whole tissue specimens obtained at diagnosis from 31 patients. Based on clinical follow-up at two years post-treatment, 11 patients showed no evidence of disease (NED), while 20 experienced disease recurrence or progression (RecPro). To assess the specificity of the identified peptide signatures to the CDDP-CRT treatment regimen, the performance of the trained classification models was evaluated in a separate cohort of 29 HPV-negative HNSCC patients, treated with mitomycin C-based chemoradiotherapy (MMC-CRT). These patients were participants of the same clinical trial and were matched with the CDDP-CRT cohort for age and disease stage. Clinicopathological characteristics of both cohorts are detailed in Table 1, whereas the consort diagram outlining patient selection is shown in Supplementary Figure S1.

**Figure 1:**
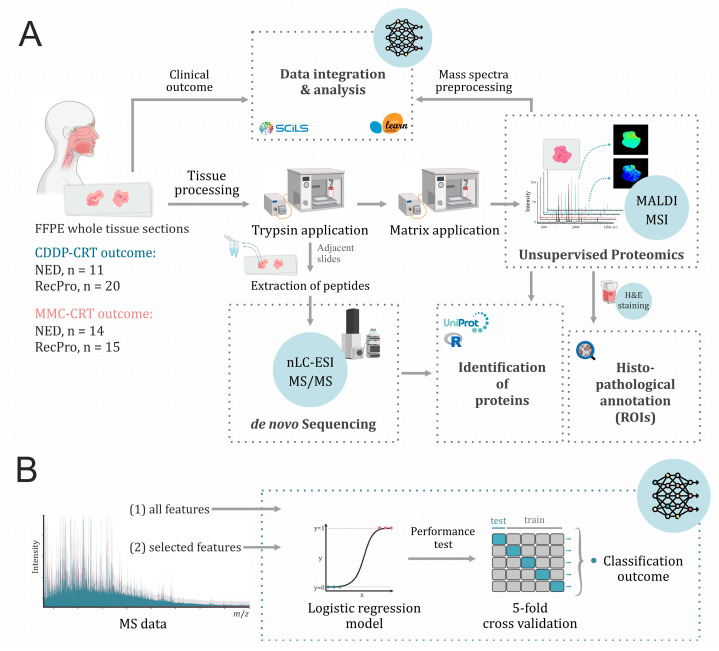
Schematic of the study’s analytical workflow, featuring (**A**) tissue processing for MALDI-MSI and nano LC-MS/MS analyses, and (**B**) machine learning-based data analysis, incorporating logistic regression and 5-fold cross-validation. Some elements of the image were created with BioRender.com.

All FFPE tissue samples were randomly arrayed on 17 indium tin oxide (ITO)-coated glass slides and processed in accordance with the workflow outlined in Figure 1. Owing to the variable presence of healthy tissue—such as squamous epithelium, subepithelial stromal tissue, and salivary glands—across the samples, the final data analysis was restricted to tumor regions only, defined as areas containing at least 70% tumor cells, annotated post-measurement by reference pathologists. H&E images of exemplary tissue specimens, along with pathological annotations of tumor regions, are shown in Supplementary Figure S3.

In total, MALDI-MSI analysis of the examined CDDP-CRT tissue regions generated 109.310 mass spectra (RecPro: 57.198; NED: 52.112), with 711 aligned *m*/*z* features detected within the tryptic peptide range of *m*/*z* 600–3200, as detailed in Supplementary Table S1. The average mass spectra of tumor regions corresponding to the RecPro and NED patient groups are presented in Supplementary Figure S4.

### 2.1. Univariate Statistics in Combination with Bottom-Up Proteomics Reveals Discriminative m/z Features Associated with Treatment Outcome in HNSCC

Since no reliable markers currently exist to predict CDDP-CRT treatment outcome in HNSCC, we first conducted univariate receiver operating characteristic (ROC) curve analysis to evaluate the ability of detected *m*/*z* features in MALDI-MSI in distinguishing tumor tissue regions from patients with good and poor treatment outcome, referred to as NED and RecPro groups, respectively. ROC curves were generated for each *m*/*z* feature based on its differential spatial distribution in tumor regions corresponding to the RecPro versus NED patient groups. The resulting area under the curve (AUC) values ranged between 0.1446 and 0.5808 and are shown in Supplementary Table S1. Among the 711 aligned *m*/*z* features, 249 showed discriminatory potential as judged by a significant AUC value (AUC < 0.3 or AUC > 0.7) and Wilcoxon Rank Sum Corrected *p*-value (<0.001).

The alignment of MALDI-MSI and nano-LC-ESI-MS/MS output enabled the initial identification of 247 out of 249 discriminative *m*/*z* features revealed by ROC analyses (Supplementary Table S2, Full Peptide Library). Following well-accepted guidelines, these identifications underwent further validation, ensuring that each identified protein was supported by the detection of at least two corresponding peptide signals by MALDI-MSI with similar tissue distribution [27,28]. While a total of 33 proteins met the initial criterion (≥2 corresponding peptides; Supplementary Table S2, Short Peptide Library), consistent signal intensity distribution of the fragment peptides was confirmed for twelve proteins, listed in Supplementary Table S3 along with their supporting peptides and AUC values derived from the RecPro vs. NED ROC curve analyses. The tentatively identified proteins included actin, cytoplasmic 2 (ACTG1), catenin alpha-1 (CTNNA1), catenin beta-1 (CTNNB1), heat shock protein beta-1 (HSPB1), keratin, type I cytoskeletal 19 (KRT19), keratin, type II cytoskeletal 6A (KRT6A), myosin-9 (MYH9), myosin light polypeptide 6 (MYL6), neuroblast differentiation-associated protein (AHNAK), plectin (PLEC), sodium/potassium-transporting ATPase subunit alpha-1 (ATP1A1), and tubulin beta chain (TUBB). Each of these proteins displayed discriminatory potential, as indicated by ROC-AUC values below 0.3 for their corresponding peptides in the RecPro vs. NED analysis. This reflects a lower intensity distribution in tumor regions of the RecPro group compared to the NED group. Notably, actin, cytoplasmic 2 demonstrated the highest discriminatory power, with its corresponding peptide (*m*/*z* 1198.739) achieving an ROC-AUC value of 0.1917.

**Figure 2:**
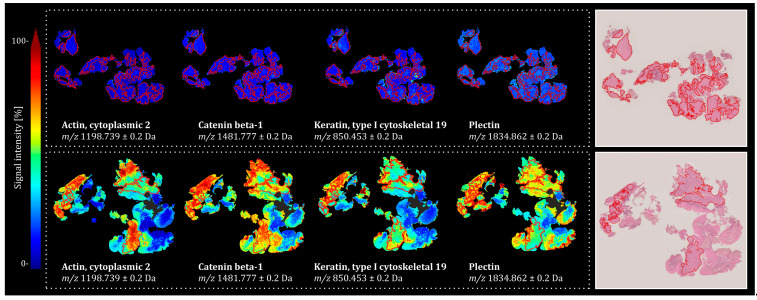
Signal intensity distribution of four tentatively identified proteins across two representative tissue specimens from the investigated cohorts: (**top**) RecPro, patient ID 362, and (**bottom**) NED, patient ID 229. Corresponding H&E-stained images are shown for each tissue, with tumor regions annotated in red.

The spatial distribution of peptides corresponding to the four most discriminatory proteins, as determined by the ROC-AUC values of their respective peptide signals, in representative tissue specimens from both groups, is presented in Figure 2. Notably, while tissue specimens from the NED group showed an overall higher signal intensity for the identified proteins, the spatial distribution of these signals was highly heterogeneous. Importantly, this variability was observed not only between tumor regions (marked in red) and the surrounding non-tumor tissue but also within the tumor regions themselves, underscoring the presence of significant intratumoral heterogeneity.

Although the identified proteins demonstrated strong discriminatory power in distinguishing treatment outcomes within the investigated tissue cohort, their effectiveness as single markers in clinical settings may be limited due to biological variability in the general population, heterogeneous expression patterns across patients, and potential confounding factors that influence marker expression [29].

### 2.2. Predictive Value of Multivariate MALDI-MSI–Derived Proteomic Signatures for Two-Year Outcomes in CDDP-CRT-Treated HNSCC

Given that molecular signatures may offer improved robustness over individual markers, we then investigated the classification capacity of the in situ proteomic landscape of HNSCC tumor resections acquired via MALDI-MSI using a multivariate modeling approach. To this end, a logistic regression model was applied to two feature sets: all detected *m*/*z* features and a curated panel of 249 discriminatory *m*/*z* features identified through ROC curve analysis, as detailed in the previous section and Supplementary Table S1. Model performance for both feature sets was assessed using five-fold cross-validation, with balanced accuracy (b.acc.), sensitivity (i.e., the ability to correctly identify positive cases—here the NED patients), and specificity (i.e., the ability to correctly identify negative cases—here the RecPro patients) serving as the primary classification metrics at both the spectral and patient levels. Performance metrics for logistic regression models trained on all MALDI-MSI *m*/*z* features and the top 249 discriminatory *m*/*z* features are summarized in Table 2. For each model, classification results are reported for individual validation splits, along with the mean performance obtained by averaging the outcomes of five trained models.

**Figure 3:**
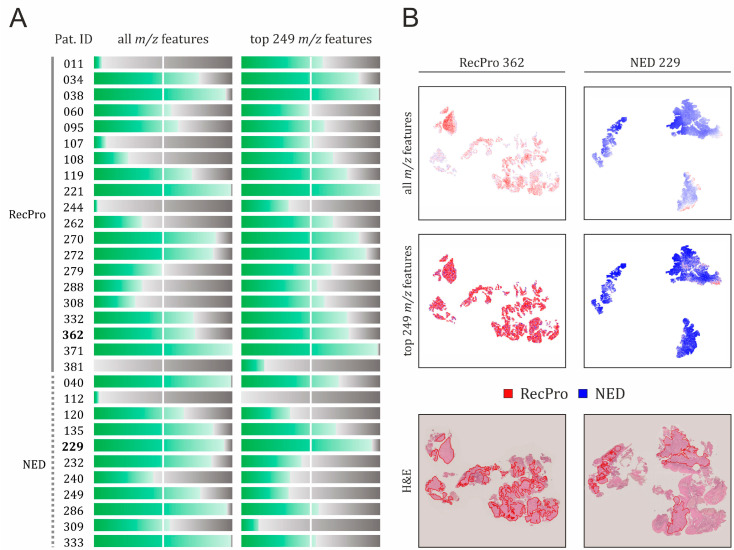
Classification performance of the logistic regression models evaluated in this study. (**A**) Performance of models trained on all *m*/*z* features and a subset of 249 *m*/*z* features with the highest discriminatory potential, as determined by the ROC-AUC analysis for RecPro vs. NED. Green bars indicate the proportion of correctly classified spectra for each patient. (**B**) Spatial distribution of class probabilities across tumor regions in both models, visualized in two representative tissue specimens. Spectra-level predictions are color-coded: red for RecPro and blue for NED, with color intensity corresponding to prediction confidence, i.e., brighter areas indicate higher certainty. Corresponding H&E-stained images with tumor regions outlined in red are shown for reference.

A graphical summary of the classification performance of both models across all patients is presented in Figure 3, alongside the spatial distribution of predicted class probabilities in two representative tissue specimens from the RecPro and NED patient groups. For spatial maps of predicted class probabilities across all 31 patients included in the study, refer to Supplementary Figure S5.

#### 2.2.1. Classification Performance Based on All MALDI-MSI *m*/*z* Features

At the spectral level, the model fitted to all *m*/*z* features detected in MALDI-MSI exhibited considerable variability across the five data splits (see Table 2). On average, it achieved a balanced accuracy of 65%, with a sensitivity of 69.3% and a specificity of 61.6%. At the patient level, all *m*/*z*-based classification models demonstrated a modest improvement in overall performance, with an average balanced accuracy of 70.5%. The corresponding mean sensitivity and specificity were 75% and 66%, respectively. However, it is important to note that the size of the test set at the patient level was necessarily small due to the limited cohort size (n = 31), leading to substantial variability across splits. Although cross-validation averaging helps mitigate some of this fluctuation, the final scores should be interpreted with caution and considered only as rough indicators of model performance at the patient level.

Based on a majority voting approach, defined as correct classification of more than 50% of all spectra per patient, the classification model trained on all *m*/*z* features correctly assigned 20 out of 31 CDDP-CRT-treated patients to their respective treatment outcome groups, as illustrated in Figure 3. Of these, 11 patients belong to the RecPro group and 9 to the NED group. In 13 of these 20 cases, the model demonstrated high classification accuracy, correctly predicting the outcome for the vast majority of nearly all spectra (75–100%). This subset included 6 RecPro patients (IDs: 034, 038, 221, 270, 272, 371) and 7 NED patients (IDs: 040, 135, 229, 232, 249, 286, 333). Conversely, 11 patients were misclassified, defined as having fewer than 50% of their spectra correctly classified by the all *m*/*z*-based model. This group included 9 patients from the RecPro group (IDs: 011, 107, 108, 244, 262, 279, 288, 308, 381) and 2 from the NED group (IDs: 112, 240).

Notably, as shown in Supplementary Figure S5, substantial intratumoral heterogeneity in in situ peptide profiles was observed in 10 patients (IDs: 060, 095, 108, 120, 240, 262, 279, 288, 308, 309), for which the classification accuracy of the all *m*/*z*-based model ranged from 25% to 65%. This variability suggests the presence of spatially distinct molecular subregions within tumors, which may complicate prognostic assessment in clinical settings—particularly when only limited tissue material is available.

The distribution and magnitude of coefficients from a model fitted to all *m*/*z* features across all five splits are presented in Supplementary Figure S6. Across the five cross-validation splits, we identified six features—*m*/*z* 628, *m*/*z* 810, *m*/*z* 944, *m*/*z* 1095, *m*/*z* 1111, and *m*/*z* 1391—which appeared adequately in at least two splits (for the complete list of model coefficients fitted across all cross-validation splits, see Supplementary Table S4). While the magnitude of these coefficients varied across splits, their directionality remained consistent, as each coefficient exhibited only positive or only negative values across all splits, underscoring the robustness of the model in feature selection. Among them, five coefficients (*m*/*z* 628, *m*/*z* 810, *m*/*z* 1095, *m*/*z* 1111, and *m*/*z* 1391) demonstrated negative mean values, suggesting association with the RecPro group, while one coefficient (*m*/*z* 944) had a positive mean value, implying a relationship with the NED group. The distribution and relative magnitudes of the key model coefficients are illustrated in Supplementary Figure S6B. Based on the parallel bottom-up proteomic analysis described in subsection B, four of these key coefficients were tentatively assigned to the following proteins: keratin, type II cytoskeletal 5 (KRT5, *m*/*z* 810.405), heterogeneous nuclear ribonucleoprotein H3 (HNRNPH3, *m*/*z* 944.499), plasminogen activator inhibitor 1 RNA-binding protein (SERBP1, *m*/*z* 1111.592), and drebrin-like protein (DBNL, *m*/*z* 1391.781), as detailed in Supplementary Table S4.

#### 2.2.2. Feature Selection as a Means to Improve Specificity of the Classification Model

Since the complete MALDI-MSI dataset—comprising all detected *m*/*z* features—may include not only relevant signals but also redundant or non-informative ones that could negatively impact patient classification, we then evaluated the performance of a logistic regression model trained on a refined subset of *m*/*z* features. This subset comprised 249 discriminatory features identified through univariate statistical testing based on the RecPro vs. NED ROC curve analysis, as described in the previous section.

As summarized in the bottom panel of Table 2, the logistic regression model based on a refined set of 249 discriminatory *m*/*z* features demonstrated substantially higher specificity compared to the model trained on all *m*/*z* features (top panel of Table 2), indicating a greater ability to avoid false positives—that is, misclassifying patients with poor outcomes as having good outcomes. Across splits, specificity ranged from 53% to 86% at the spectral level and from 75% to 100% at the patient level. On average, the model achieved specificities of 73% and 91% at the spectral and patient levels, respectively, markedly outperforming the 61.6% and 66% achieved by the full-feature model.

However, this improvement in specificity came at the cost of reduced sensitivity, reflecting a decreased ability to correctly identify true positives (here, NED patients). Compared to the full-feature model, the feature-restricted model showed a decrease in mean sensitivity; from 69% to 47% at the spectral level and from 75% to 52% at the patient level. In terms of balanced accuracy, the feature-restricted model performed slightly worse than the original one at the spectral level, achieving on average 60% compared to 65%. At the patient level, performance of both models was similar, with average balanced accuracy of 70.5% being recorded for the all *m*/*z*-based model and 71.7% for the feature-restricted one.

As shown in Figure 3, based on majority voting, the model restricted to 249 *m*/*z* features correctly classified 22 patients (17 RecPro, 5 NED), while 9 were misclassified (3 RecPro, 6 NED). High classification accuracy (75–100%) was achieved in 10 patients (IDs: 034, 038, 119, 221, 229, 270, 272, 332, 362, 371), among whom all but one (ID 229) belonged to the RecPro group.

Compared to the model trained on all *m*/*z* features, the model based on the 249 selected *m*/*z* features demonstrated improved classification accuracy in 16 patients (IDs: 011, 034, 038, 107, 108, 119, 221, 244, 262, 272, 279, 288, 308, 332, 362, 381), all of whom belonged to the RecPro group. Notably, in several of these patients (IDs: 011, 107, 108, 262, 279, 288, 308), accuracy improved substantially, shifting them from misclassified (accuracy ≤ 50%) to correctly classified (accuracy > 50%). In contrast, a decrease in classification accuracy was observed in 13 patients (IDs: 040, 060, 112, 120, 135, 232, 240, 249, 270, 286, 309, 333, 371), the majority of whom (10 out of 13) belonged to the NED group. Among these, four NED patients (IDs: 120, 232, 249, 309) transitioned from correct to incorrect classification. Within the RecPro group, only one patient (ID: 060) experienced a decline in classification accuracy that led to misclassification. Finally, classification accuracy remained unchanged in two patients (IDs: 095 and 229), one from each clinical group (RecPro and NED, respectively).

As visualized in Supplementary Figure S5, in 16 patients (IDs: 011, 060, 095, 107, 108, 120, 232, 240, 244, 249, 262, 279, 286, 288, 308, 333), considerable intratumoral heterogeneity was observed based on the recorded peptide profiles, with classification accuracies ranging from 36% to 67%. Despite this variability, the majority (10 patients: 011, 095, 107, 108, 262, 279, 286, 288, 308, 333) were still correctly classified based on majority voting. However, six patients (IDs: 060, 120, 232, 240, 244, 249) were misclassified.

Yet again, this degree of heterogeneity underscores the challenge of reliably assessing patient outcomes in a clinical setting, particularly when analyses are based on limited or unrepresentative tumor samples.

### 2.3. Performance of Classification Models in HNSCC Patient Cohort Subjected to Other Treatment Regimens

To assess whether the identified peptide signatures and classification models are specific to prognosing outcomes following CDDP-CRT, we evaluated their performance in another HNSCC patient cohort that received mitomycin C-based CRT instead of the platinum-based regimen. This cohort originated from the same clinical study and was matched to the CDDP-CRT group by age and HPV status. Tissue specimens from the MMC-CRT cohort were randomly deposited across the same 17 glass slides that were used for the CDDP-CRT cohort to minimize potential batch effects. Clinicopathological details of the MMC-CRT cohort are presented in Table 1. The performance of the two classification models, at both the spectra and patient levels, is summarized in Table 3.

The classification models trained on the CDDP-CRT-treated HNSCC patient cohort performed poorly when applied to the MMC-CRT cohort. At the spectra level, balanced accuracy across cross-validation splits did not exceed 57% for the model using all *m*/*z* features and 53% for the model restricted to the top 249 *m*/*z* features. Performance was even worse at the patient level, with balanced accuracy rarely surpassing 50%. Both models consistently exhibited very low sensitivity across all splits at both the spectra and patient classification levels in the MMC-CRT cohort. In contrast, the specificity of the all *m*/*z* model in the MMC-CRT cohort was comparable to its performance in the CDDP-CRT cohort. However, the 249 *m*/*z* feature model showed a notable decline in specificity in the MMC-CRT cohort at both classification levels.

## 3. Discussion

Despite advances in therapeutic strategies, HNSCC patients exhibit high recurrence, highlighting the medical need for robust molecular signatures to improve the patient risk assessment and enable personalized treatment.

In this study, we employed MALDI-MSI of tryptic peptides to assess whether the spatially resolved intrinsic proteomic profiles of tumors resected from advanced-stage HNSCC patients—prior to the initiation of platinum-based chemoradiotherapy—could provide prognostic insights into treatment outcomes evaluated two years post-therapy. Patients were stratified based on clinical follow-up into two groups: poor outcome (RecPro) and favorable outcome (NED).

Intratumoral heterogeneity is a major contributor to treatment failure, drug resistance, and tumor recurrence, making it difficult to identify robust molecular signatures for accurate risk stratification [30,31]. Despite advances, the comprehensive understanding of how intratumoral heterogeneity and proteomic profiles affect HNSCC remains limited. This limitation underscores the need for integrative methods that combine spatial molecular analysis with histology to better understand the characterization of tumor behavior and guide therapeutic intervention. Given that tissue microarrays (TMAs) offer only a restricted view of tumor complexity and heterogeneity due to their limited size [32], we purposely analyzed whole tissue sections in this study. Furthermore, our analysis was restricted to tumor regions within the investigated HNSCC tissue specimens, as the prognostic relevance of specific molecular features in HNSCC can vary across distinct tissue compartments [33].

As part of a standard data analysis pipeline for MALDI-MSI datasets, we first analyzed the recorded peptide signatures using univariate statistical methods (ROC) to evaluate the discriminatory potential of individual *m*/*z* values distinguishing the two patient groups, RecPro and NED. By integrating these results with complementary bottom-up proteomics using nano-LC-MS/MS, twelve proteins were assigned to the corresponding *m*/*z* values exhibiting elevated signal intensities in patients with a favorable CDDP-CRT outcome (NED group) compared to patients with less favorable outcomes (RecPro). The majority of these proteins are structural components of the cytoskeleton and are involved in molecular function and cell adhesion. These include keratin 19, keratin 6A, plectin, actin cytoplasmic 2, tubulin beta chain, myosin light polypeptide 6, myosin heavy chain 9, heat shock protein beta-1, catenin alpha-1, catenin beta-1, integrin beta-4, and neuroblast differentiation-associated protein AHNAK [34,35,36,37,38,39,40].

Although the role of these proteins in modulating CRT outcome in HNSCC remains largely unexplored, altered expression of some has already been implicated in head and neck cancer development and progression. For example, downregulation of keratin 19 has been shown to influence the invasive phenotype of several squamous cell carcinoma cell lines, including those derived from the oral epithelium [41]. AHNAK has been reported to function as a tumor suppressor in nasopharyngeal carcinoma, where its downregulation was shown to be associated with poor overall survival. Consecutively, silencing AHNAK in both in vitro and in vivo settings has been shown to enhance the growth, invasion, and metastatic potential of NPC cells [42]. Overexpression of plectin in HNSCC compared to adjacent non-cancerous tissue has been well documented and suggested to promote cancer cell migration and invasion, as suppression of endogenous plectin has been shown to inhibit these processes and downregulate ERK1/2 kinase [43]. However, other studies indicate that ERK1/2 signaling plays a protective role in HNSCC, particularly in resistance to cetuximab and fractionated IR treatment [44]. It remains to be elucidated whether the increased plectin signal observed among patients responding favorably to CRT treatment (which, as a combinatory treatment, does involve radiation) in our study is linked to the protective effects of ERK1/2 activation. It should be noted that MALDI-MSI alone cannot reveal the molecular pathways underlying the observed proteomic alterations in tissue. However, it can serve as a valuable tool to guide more detailed, albeit lower-throughput, proteomic analyses of specific regions of interest using complementary techniques such as laser-capture microdissection (LCM) [45].

In addition to spatial context, molecular signatures—combinations of multiple markers—have demonstrated better reliability for patient stratification and disease classification compared to single molecular markers, whose interpretative value is often limited by interindividual variability [46,47,48,49].

MALDI-MSI provides spatially resolved molecular signatures, making it particularly well-suited for characterizing complex molecular patterns in diseased tissue. However, the high dimensionality of the data presents significant analytical challenges—especially when working with complex biological samples such as whole tissue specimens, as in our study—requiring both intensive mathematical and computational processing. Notably, the rich spatial and spectral information captured by MALDI-MSI is well-suited for machine learning (ML) approaches, which can effectively extract meaningful features from molecular distributions and spectral intensities [24].

In this study, we implemented logistic regression models to evaluate the classification performance of intrinsic peptide profiles of investigated HNSCC tumors in distinguishing patients showing distinct treatment outcomes at 2 years post-treatment. Since the performance of the classification model may vary depending on the number of features being used [50], we assessed the performance of two models: one trained on the full, uncurated set of *m*/*z* features detected by MALDI-MSI in tissue samples of the CDDP-CRT cohort, and another trained on a curated subset of features that demonstrated discriminatory potential in univariate ROC curve analysis, in total 249 *m*/*z* features. Our aim was to investigate whether the selection and composition of input features influence the performance of the classification models.

By integrating ML with full MALDI-MSI-derived in situ peptide profiles—using all detected *m*/*z* features—the model achieved on average a balanced accuracy of 70% at the patient level, with 75% sensitivity and 66% specificity in classifying HNSCC patients based on their CRT treatment outcomes. While this performance may seem modest statistically, it is noteworthy given the substantial inter- and intra-individual variability inherent in biological materials [51,52]. Notably, the all features-based model demonstrated good performance in identifying true positives, specifically patients with a favorable treatment outcome, defined as NED, two years post-treatment.

In our cohort, feature selection improved model specificity to nearly 92% at the patient level, significantly reducing false positives and thereby enhancing the identification of patients likely to experience poor outcomes from CDDP-CRT (i.e., the RecPro group). Accurate prognostication of an unfavorable outcome is particularly crucial, as CRT is associated with considerable cellular toxicity, which can elevate the risk of non-cancer-related mortality [7].

However, the gain in specificity in the feature-restricted classification model came at the cost of reduced sensitivity, reflecting a higher rate of false negatives—i.e., misclassifying patients who are actually likely to experience a good treatment outcome. This trade-off was clearly evident in the NED group, where several patients shifted from being correctly to incorrectly classified following feature selection. These findings highlight a critical consideration in clinical model optimization: while feature selection can enhance classification performance for one subgroup (in this case, RecPro), it may reduce accuracy for others. It is important to note that the reduced sensitivity of the feature-restricted model in our study is likely attributable to the small cohort size and pronounced group imbalance, particularly the underrepresentation of patients with favorable treatment outcomes (i.e., the NED group, n = 11). The resulting limited biological variability could have contributed to model underfitting, affecting the robustness and generalizability of the results. Future studies involving larger and more balanced cohorts will be crucial to validate our findings and more fully capture patient heterogeneity and biological variability, ultimately enhancing model performance and clinical applicability.

In addition to evaluating the classification performance of in situ proteomic profiles for prognosticating outcomes of CDDP-CRT, we also assessed whether the generated models could identify patients likely to respond well or poorly to an alternative chemoradiotherapy regimen—MMC-CRT. Interestingly, both models demonstrated poor performance in this setting, suggesting that the identified peptide-based proteomic signatures may be specifically associated with response to platinum-based treatment. As such, the results of this study should not be generalized to other CRT regimens. Moreover, due to the relatively small sample size, the presented findings warrant validation in a larger, independent cohort.

Although, to the best of our knowledge, this is the very first study implementing MALDI-MSI to investigate the capacity of the intrinsic HNSCC proteome profile in distinguishing CRT treatment outcome, this technique has already been utilized to explore molecular signatures in HNSCC in the past. The majority of studies have focused on comparing the molecular composition of tumors with normal tissue or the surrounding microenvironment, underscoring its role in diagnostic tumor classification. Despite these differing objectives, the findings of these studies may still provide valuable insights in the context of our research.

Bednarczyk et al. [53] applied MALDI-MSI to analyze proteomic and lipidomic profiles in cryopreserved HNSCC tissue from the tongue, comparing cancerous epithelium to normal mucosa. Notably, protein-based signatures outperformed lipid-based signatures in tissue classification and showed greater regional variation in signal abundance, emphasizing the potential of these biomolecules for deciphering the complexity of HNSCC. Hoffmann et al. [21] also used MALDI-MSI to profile tumor and non-tumor regions in fresh-frozen and FFPE HNSCC samples. Among tumor-specific signals reported in this study, two aligned with key coefficients in our classification model (*m*/*z* 944 and *m*/*z* 1095). Additionally, similar protein families, i.e., keratins and heat shock proteins, were identified as discriminatory in both studies, reinforcing the reproducibility and clinical relevance of these markers for HNSCC.

Even more directly relevant to our own research, Kurczyk et al. [22] employed MALDI-MSI to evaluate the prognostic significance of ITH in oral cancer and its lymphatic metastases, similarly assessed at the level of tryptic peptides. Interestingly, the study demonstrated that ITH is positively associated with disease outcome. Specifically, greater intra-tumor similarity in peptidomic spectra was associated with poorer prognosis, whereas higher levels of ITH, thought to reflect the presence of heterotypic components within the tumor microenvironment, such as infiltrating immune cells, were observed in patients with more favorable outcomes. Although we could not confirm this particular relationship in our HNSCC cohort, we did observe substantial heterogeneity within the investigated HNSCC tumor regions, which appeared homogeneous under conventional H&E staining. Furthermore, while not the focus of our study, we also observed a high degree of heterogeneity in the tumor surrounding, suggesting that spatially resolved proteomic analysis of the tumor microenvironment, including tumor–stroma interface, could likewise yield biologically significant insights and represent a promising avenue for future research.

These observations further underline the importance of spatially resolved analyses, since these ITH profiles would be irretrievably lost during homogenization of the tissue, which is a prerequisite for all tissue bulk analysis techniques [54]. While our whole-slide analysis enabled assessment of ITH at the biopsied site, a more comprehensive characterization could be achieved in future studies by analyzing multiple regions within the same tumor. We acknowledge, however, that multi-region sampling of whole-tissue specimens would considerably increase acquisition time and data volume, thereby posing practical challenges for throughput and scalability. With ongoing technological advancements, we anticipate that large-scale, multi-region analyses utilizing larger and more representative tissue specimens will become increasingly feasible, thereby enabling a more comprehensive understanding of spatial heterogeneity in these tumors.

In conclusion, in this study, MALDI-MSI in combination with ML was able to identify patients at elevated risk of recurrence following CDDP-CRT. In addition to facilitating tissue classification, these profiles show promise as prognostic tools, addressing a current gap in biomarker availability for this clinically and biologically heterogeneous group of malignancies. Given the substantial interpatient variability inherent to HNSCC, the prognostic performance of the proposed classification models must be validated in larger patient cohorts. This is expected to enhance classification sensitivity, an essential requirement for the reliable identification of treatment responders, and may provide deeper insights into the relationship between intratumoral heterogeneity and response to CRT.

## 4. Materials and Methods

### 4.1. Patient Material

Formalin-fixed, paraffin-embedded (FFPE) primary tumor specimens were obtained at diagnosis from 60 HNSCC patients enrolled in the ARO-0401 phase III clinical trial. ARO-0401 was a prospective, randomized, multicentre study (total n = 364; recruitment completed in 2008) designed to optimize concurrent chemoradiotherapy (CRT) for advanced oropharyngeal and hypopharyngeal carcinoma [55]. A consort flowchart detailing the patient selection process for MALDI-MSI analysis is presented in Supplementary Figure S1, whereas Kaplan–Meier curves comparing overall survival between included and non-included patients are presented in Supplementary Figure S2. All patients in the MALDI-MSI cohort had unresectable, locally advanced stage IVAB disease of the oropharynx or hypopharynx and were treated with hyperfractionated, accelerated radiotherapy combined with either 5-fluorouracil (5-FU) and cis-dichloro-diammin-platinum II (CDDP-CRT, n = 31) or 5-FU and mitomycin C (MMC-CRT, n = 29). Tissue collection followed the same protocol and time frame for both cohorts. Based on the outcome evaluated two years post-treatment, patients were categorized into two groups, namely no evidence of disease (NED) and recurrence/progression (RecPro). In the NED groups, no disease recurrence was observed during the follow-up period, with a median ± standard deviation of 62 ± 21 months for the CDDP-CRT cohort and 61 ± 23 months for the MMC-CRT cohort. Human papillomavirus (HPV) status was negative in all patients, as confirmed by both p16 immunohistochemistry and PCR-based HPV DNA testing, as previously described [56]. The MMC-CRT cohort was included to determine whether peptide profiles identified in the CDDP-CRT cohort are specific to platinum-based therapy. The clinicopathological characteristics of the investigated patient cohorts are summarized in Table 1. The study was approved by the local Ethics Committee (vote number EA2/086/10). Informed patient consent was obtained for the tumor samples used in the study.

### 4.2. Tissue Sample Preparation and MALDI-MSI Measurement

MALDI-MSI was conducted on 5 µm thick FFPE tissue sections mounted on conductive glass slides (MALDI IntelliSlides, Bruker Daltonik GmbH, Bremen, Germany). A total of 17 glass slides were used, with tissue specimens from all 60 investigated patients (31 CDDP-CRT, 29 MMC-CRT) being randomly distributed across the slides. The slides were incubated at 60 °C for 1 h, then deparaffinized and rehydrated through sequential washing in xylene (2 × 5 min), isopropanol (100%; 1 × 5 min), graded ethanol (100%, 96%, 70%, and 50%; 1 × 5 min each), and MilliQ water (2 × 10 s). To reverse formaldehyde-induced protein cross-linking, heat-induced antigen retrieval was performed in near-boiling MilliQ water (~98 °C) for 20 min in a steamer. Afterwards, the slides were scanned at high resolution using a digital slide scanner (Reflecta MF 5000; Reflecta, Eutingen im Gäu, Germany). Trypsin/LysC solution (0.025 µg/µL in 20 mM ammonium bicarbonate buffer with 0.01% glycerol; Promega, Madison, WI, USA) was evenly applied onto the tissue sections using an automated spraying device (HTX TM-sprayer, HTX Technologies, Chapel Hill, NC, USA) under the following conditions: 16 layers, flow rate of 0.015 mL/min, spray velocity of 750 mm/min, track spacing of 2 mm, and nozzle temperature set to 30 °C. Enzymatic digestion was performed at 50 °C for 2 h in a humidified chamber containing saturated potassium sulfate solution. The slides were then coated with α-cyano-4-hydroxycinnamic acid (CHCA) matrix solution (7 mg/mL in 70% ACN, 1% TFA; Bruker Daltonik GmbH) using the same spraying device, with the following parameters: 4 layers, flow rate of 0.120 mL/min, velocity of 1200 mm/min, track spacing of 3 mm, and nozzle temperature of 75 °C. MALDI-MSI measurements were performed on a rapifleX MALDI Tissuetyper (Bruker Daltonik GmbH) mass spectrometer operating in positive ionization reflectron mode at a spatial resolution of 50 µm. The mass range was set to 600–3200 *m*/*z*, with 500 laser shots per spot and a sampling rate of 1.25 GS/s. MSI data were acquired using flexImaging 5.1 and flexControl 3.0 software (Bruker Daltonik GmbH). External calibration was performed using the Peptide Calibration Standard II (0.5 μL; PepMix; Bruker Daltonik GmbH), manually spotted in tissue-free areas of each slide before measurement. Following data acquisition, the CHCA matrix was removed by washing with 70% ethanol (2 × 2 min) and MilliQ water (1 × 1 min). The tissue sections were stained with hematoxylin and eosin (H&E) for histological annotation and scanned at 20× magnification using a digital slide scanner (NanoZoomer-SQ, Hamamatsu Photonics K.K., Hamamatsu City, Japan).

The annotation of tumor regions, defined as areas containing at least 70% tumor cells, was performed by two pathologists according to the “four-eyes” principle. To minimize temporal variability and ensure consistency across the dataset, all slides were annotated on the same day under identical conditions. Exemplary images of H&E-stained tissue specimens, along with pathological annotations of tumor regions, are provided in Supplementary Figure S3.

### 4.3. MALDI-MSI Data Processing

MALDI-MSI raw data were imported into and processed in SCiLS Lab 2024a Pro software (Bruker Daltonik GmbH, Bremen, Germany). Baseline removal was performed using the TopHat algorithm, with a peak width of 200, followed by total ion count normalization. Regions of interest (ROIs) corresponding to tumor areas were annotated using QuPath software under the guidance of a trained pathologist (S.S.) from the Institute of Pathology, Charité – Universitätsmedizin Berlin [57]. The H&E-stained images of each tissue section, along with the corresponding ROIs, were co-registered with their respective MSI measurement data. The dataset was divided into distinct subsets, each containing mass spectral data specific to one of the patient groups under investigation: NED and RecPro. Peak detection and alignment were performed on the subset corresponding to the annotated tumor regions, utilizing a standard segmentation pipeline in SCiLS Lab. This pipeline applied TIC normalization, medium denoising, and peak interval width adjustment to ±0.200 Da for peak finding and spectral alignment.

### 4.4. Univariate Statistical Testing

Receiver operating characteristic (ROC) curve analysis was conducted in SCiLS Lab software for all aligned *m*/*z* features to assess their ability to discriminate between HNSCC patients with differing treatment responses (RecPro vs. NED). To account for variations in tissue size, ROC curve analyses were performed on a defined subset of mass spectra, ensuring an equal number of spectra for both comparison groups. Classification performance was considered significant when the AUC was either above 0.7 or below 0.3, the latter accounting for potential inverse correlations [58,59]. Values between 0.3 and 0.7 were not considered discriminative.

### 4.5. Machine Learning-Based Data Analysis

Machine learning analysis, including logistic regression and cross-validation, was conducted outside of the SCiLS Lab software environment using the scikit-learn open-source tool (https://scikit-learn.org; accessed on 15 May 2025). For this purpose, the acquired mass spectra, along with their corresponding XY coordinates, were exported from SCiLS Lab as .csv files.

Logistic regression analyses were performed in two ways: (1) using all detected *m*/*z* features, with each spectrum down-sampled by a factor of 2× by averaging the signal at every second *m*/*z* value, and (2) using a selected subset of 249 *m*/*z* features identified as discriminatory based on ROC analysis. The logistic regression models were regularized using an L1 penalty to encourage sparse solutions. The L1 regularization parameters were optimized to enhance model performance. For all detected *m*/*z* values, we used a regularization strength of 7000, and for the subset of 249 *m*/*z* features, we used a strength of 30. To account for class imbalance, the classes were weighted inversely proportional to their frequency during model fitting. Model performance was evaluated using 5-fold cross-validation with class-stratified sampling, ensuring that spectra from the same patient appeared only in either the training or test set within each fold, but never in both. To account for systemic differences between patients, spectra from each patient were additionally normalized with the mean and standard deviation spectrum computed over the given patient. Sensitivity, specificity, and balanced accuracy (b.acc.), defined as the arithmetic mean of sensitivity and specificity, together with the ROC-AUC values, were chosen as the primary metrics to assess classification performance. The model performance metrics were evaluated at two levels: the spectra level and the patient level. Spectra-level metrics reflect the overall balanced accuracy across all spectra, while patient-level metrics were derived by summing the predicted class probabilities for all spectra from a given patient. The final patient classification was assigned to the class with the highest aggregated probability. For each model, we report classification performance on individual data splits, as well as the mean performance, obtained by averaging results across five splits.

### 4.6. Protein Identification by Nano-LC-ESI-MS/MS

A bottom-up nano-liquid chromatography electrospray ionization tandem mass spectrometry (nano-LC-ESI-MS/MS) analysis was performed on serial tissue sections from 4 randomly selected HPV/p16-negative HNSCC patients to identify *m*/*z* features detected by MALDI-MSI. The slides were processed according to the MALDI-MSI workflow up to the enzymatic digestion step. The generated peptides were then extracted from the tissue surface using 100 µL of 0.1% TFA solution at room temperature and purified with ZipTip C18 pipette tips (Merck KGaA, Darmstadt, Germany) following the manufacturer’s protocol. After drying using a vacuum centrifuge, the peptides were reconstituted in 20 µL of 0.1% TFA and stored at −20 °C until further analysis. For nano-LC-ESI-MS/MS measurement, 2 µL of peptide eluate was injected into a Dionex Ultimate 3000 nano-HPLC system (Thermo Fisher Scientific) coupled to an ESI-QTOF ultrahigh-resolution mass spectrometer (Impact II, Bruker Daltonik GmbH, Bremen, Germany). Peptide analytes were first loaded onto an Acclaim PepMap 100 C18 trap column (100 µm × 2 cm, Thermo Fisher Scientific) in 0.1% TFA, followed by separation on an Acclaim PepMap RSLC C18 analytical column (75 µm × 50 cm, Thermo Fisher Scientific). The peptide separation was performed over a 90 min gradient, with the acetonitrile (ACN) concentration increasing from 2% to 35% in 0.1% formic acid (FA), at a flow rate of 400 nL/min. The system operated within a pressure range of 10–800 bar, and the column temperature was maintained at 60 °C. Internal calibration was achieved using a 10 mM sodium hypofluorite solution at a flow rate of 20 µL/h.

Data acquisition was performed using a data-dependent acquisition method, with precursor ions automatically selected for MS/MS fragmentation via collision-induced dissociation using Auto MS/MS InstantExpertise mode. The raw data files (.baf) were analyzed using PEAKS Studio 12 (Bioinformatics Solutions Inc., Waterloo, ON, Canada) [60]. Peptide searches were conducted against the human UniProtKB/Swiss-Prot database using the following parameters: precursor ion mass tolerance of 20.0 ppm and fragment ion mass tolerance of 0.05 Da; trypsin as the enzyme with up to two missed cleavages allowed; a maximum of two variable post-translational modifications per peptide, including methionine oxidation (+15.99 Da) and N-terminal protein acetylation (+42.01 Da). The false discovery rate was controlled at 1%, and protein identification required at least one unique peptide. Only the first identification (ID) with the highest −logP score for each protein group was used for the subsequent data evaluation.

Finally, in order to identify MALDI-MSI *m*/*z* values, peptides with the smallest mass deviation and highest −logP values were matched to the generated LC–MS/MS database using custom R scripts developed in-house.

## Figures and Tables

**Table 1:** Clinicopathological characteristics of the investigated patient cohorts.

		CDDP-CRT	MMC-CRT
	total number (n)	31	29
Age at diagnosis mean (±SD)		56.4 (±9.3)	56.1 (±6.2)
Sex	male	24 (77%)	23 (79%)
	female	7 (23%)	6 (21%)
HPV	negative	33 (100%)	29 (100%)
Stage of the disease	IVA	27 (87%)	26 (90%)
	IVB	4 (13%)	3 (10%)
Risk category ^1^	high	33 (100%)	29 (100%)
Smoking	yes	18 (58%)	23 (79%)
	no	13 (42%)	6 (21%)
Localization	oropharynx	15 (55%)	16 (55%)
	hypopharynx	16 (45%)	13 (45%)
Outcome ^2^	NED	11 (35%)	14 (48%)
	RecPro	20 (65%)	15 (52%)

^1^ Risk category assessed according to Ang K. Kiang et al. [26]. ^2^ Outcome assessed at 24 months post-treatment.

**Table 2:** Classification results and model performance metrics across five cross-validation splits using the full (all *m*/*z* features) and the restricted (top 249 discriminatory *m*/*z* features) MALDI-MSI datasets.

Model	Level	Metric	Split 1	Split 2	Split 3	Split 4	Split 5	Mean
all *m*/*z* features	Spectra	b.acc.	0.917	0.388	0.824	0.690	0.452	0.654
		AUC	0.975	0.293	0.899	0.892	0.449	0.702
		sensitivity	0.945	0.049	0.830	0.993	0.646	0.693
		specificity	0.888	0.727	0.819	0.388	0.257	0.616
	Patient	b.acc.	1.000	0.375	0.900	0.750	0.500	0.705
		AUC	1.000	0.500	1.000	0.833	0.438	0.754
		sensitivity	1.000	0.000	1.000	1.000	0.750	0.750
		specificity	1.000	0.750	0.800	0.500	0.250	0.660
249 discriminatory *m*/*z* features	Spectra	b.acc.	0.903	0.414	0.679	0.544	0.471	0.602
		AUC	0.967	0.470	0.791	0.555	0.433	0.643
		sensitivity	0.947	0.033	0.489	0.554	0.345	0.474
		specificity	0.859	0.794	0.869	0.535	0.597	0.731
	Patient	b.acc.	1.000	0.500	0.667	0.917	0.500	0.717
		AUC	1.000	0.500	1.000	0.833	0.375	0.742
		sensitivity	1.000	0.000	0.333	1.000	0.250	0.517
		specificity	1.000	1.000	1.000	0.833	0.750	0.917

**Table 3:** Performance metrics of classification models trained on the CDDP-CRT cohort applied to the MMC-CRT patient cohort.

Model	Level	Metric	Split 1	Split 2	Split 3	Split 4	Split 5	Mean
all *m*/*z* features	Spectra	b.acc.	0.509	0.568	0.564	0.538	0.534	0.543
		AUC	0.524	0.586	0.585	0.575	0.546	0.563
		sensitivity	0.399	0.469	0.454	0.573	0.480	0.475
		specificity	0.618	0.667	0.674	0.504	0.589	0.610
	Patient	b.acc.	0.440	0.540	0.474	0.445	0.443	0.469
		AUC	0.476	0.476	0.538	0.467	0.457	0.483
		sensitivity	0.214	0.214	0.214	0.357	0.286	0.257
		specificity	0.667	0.867	0.733	0.533	0.600	0.680
249 discriminatory *m*/*z* features	Spectra	b.acc.	0.485	0.466	0.509	0.524	0.465	0.490
		AUC	0.477	0.460	0.506	0.526	0.440	0.482
		sensitivity	0.415	0.257	0.340	0.449	0.198	0.332
		specificity	0.555	0.675	0.679	0.599	0.733	0.648
	Patient	b.acc.	0.376	0.369	0.436	0.405	0.469	0.411
		AUC	0.438	0.390	0.424	0.419	0.448	0.424
		sensitivity	0.286	0.071	0.071	0.143	0.071	0.129
		specificity	0.467	0.667	0.800	0.667	0.867	0.693

## Data Availability

The original contributions presented in this study are included in the article/Supplementary Materials. Further inquiries can be directed to the corresponding author.

## Supplementary Materials

The supporting information can be downloaded at: https://www.mdpi.com/article/10.3390/ijms26189084/s1.

## Abbreviations

The following abbreviations are used in this manuscript:

5-FU	5-fluorouracil
AUC	Area under the curve
CDDP	cis-Diammindichloridoplatin (cisplatin)
CHCA	α-Cyano-4-hydroxycinnamic acid
CRT	Chemoradiotherapy
FFPE	Formalin-fixed paraffin-embedded
H&E	Hematoxylin and eosin
HNSCC	Head and neck squamous cell carcinoma
HPV	Human papillomavirus
ITH	Intratumoral heterogeneity
ITO	Indium tin oxide
LC	Liquid chromatography
LCM	Laser capture microdissection
MALDI	Matrix-assisted laser desorption/ionization
ML	Machine learning
MMC	Mitomycin C
MSI	Mass spectrometry imaging
MS/MS	Tandem mass spectrometry
*m*/*z*	Mass-to-charge ratio
NED	No evidence of disease
RecPro	Recurrence/progression
ROC	Receiver operating characteristic
ROI	Region of interest
TNM	Tumor, node, metastasis

## References

[1] Sung H., Ferlay J., Siegel R.L., Laversanne M., Soerjomataram I., Jemal A., Bray F. (2021). Global Cancer Statistics 2020: GLOBOCAN Estimates of Incidence and Mortality Worldwide for 36 Cancers in 185 Countries. CA Cancer J. Clin..

[2] Ferlay J., Colombet M., Soerjomataram I., Mathers C., Parkin D.M., Pineros M., Znaor A., Bray F. (2019). Estimating the global cancer incidence and mortality in 2018: GLOBOCAN sources and methods. Int. J. Cancer.

[3] Gormley M., Creaney G., Schache A., Ingarfield K., Conway D.I. (2022). Reviewing the epidemiology of head and neck cancer: Definitions, trends and risk factors. Br. Dent. J..

[4] Burri R.J., Lee N.Y. (2009). Concurrent chemotherapy and radiotherapy for head and neck cancer. Expert. Rev. Anticancer Ther..

[5] Pignon J.P., le Maitre A., Maillard E., Bourhis J., Group M.-N.C. (2009). Meta-analysis of chemotherapy in head and neck cancer (MACH-NC): An update on 93 randomised trials and 17,346 patients. Radiother. Oncol..

[6] Rettig E.M., D’Souza G. (2015). Epidemiology of head and neck cancer. Surg. Oncol. Clin. N. Am..

[7] Rivelli T.G., Mak M.P., Martins R.E., da Costa e Silva V.T., de Castro G. (2015). Cisplatin based chemoradiation late toxicities in head and neck squamous cell carcinoma patients. Discov. Med..

[8] Budach V., Tinhofer I. (2019). Novel prognostic clinical factors and biomarkers for outcome prediction in head and neck cancer: A systematic review. Lancet Oncol..

[9] Amin M.B., Greene F.L., Edge S.B., Compton C.C., Gershenwald J.E., Brookland R.K., Meyer L., Gress D.M., Byrd D.R., Winchester D.P. (2017). The Eighth Edition AJCC Cancer Staging Manual: Continuing to build a bridge from a population-based to a more "personalized" approach to cancer staging. CA Cancer J. Clin..

[10] Lydiatt W.M., Patel S.G., O’Sullivan B., Brandwein M.S., Ridge J.A., Migliacci J.C., Loomis A.M., Shah J.P. (2017). Head and Neck cancers-major changes in the American Joint Committee on cancer eighth edition cancer staging manual. CA Cancer J. Clin..

[11] Chen T.C., Wu C.T., Ko J.Y., Yang T.L., Lou P.J., Wang C.P., Chang Y.L. (2020). Clinical characteristics and treatment outcome of oropharyngeal squamous cell carcinoma in an endemic betel quid region. Sci. Rep..

[12] Mroz E.A., Tward A.D., Pickering C.R., Myers J.N., Ferris R.L., Rocco J.W. (2013). High intratumor genetic heterogeneity is related to worse outcome in patients with head and neck squamous cell carcinoma. Cancer.

[13] Mroz E.A., Tward A.D., Hammon R.J., Ren Y., Rocco J.W. (2015). Intra-tumor genetic heterogeneity and mortality in head and neck cancer: Analysis of data from the Cancer Genome Atlas. PLoS Med..

[14] Berghmans E., Boonen K., Maes E., Mertens I., Pauwels P., Baggerman G. (2020). Implementation of MALDI Mass Spectrometry Imaging in Cancer Proteomics Research: Applications and Challenges. J. Pers. Med..

[15] Schone C., Hofler H., Walch A. (2013). MALDI imaging mass spectrometry in cancer research: Combining proteomic profiling and histological evaluation. Clin. Biochem..

[16] Stillger M.N., Li M.J., Honscheid P., von Neubeck C., Foll M.C. (2024). Advancing rare cancer research by MALDI mass spectrometry imaging: Applications, challenges, and future perspectives in sarcoma. Proteomics.

[17] Klein O., Kanter F., Kulbe H., Jank P., Denkert C., Nebrich G., Schmitt W.D., Wu Z., Kunze C.A., Sehouli J. (2019). MALDI-Imaging for Classification of Epithelial Ovarian Cancer Histotypes from a Tissue Microarray Using Machine Learning Methods. Proteom. Clin. Appl..

[18] Pertzborn D., Arolt C., Ernst G., Lechtenfeld O.J., Kaesler J., Pelzel D., Guntinas-Lichius O., von Eggeling F., Hoffmann F. (2022). Multi-Class Cancer Subtyping in Salivary Gland Carcinomas with MALDI Imaging and Deep Learning. Cancers.

[19] Patterson N.H., Alabdulkarim B., Lazaris A., Thomas A., Marcinkiewicz M.M., Gao Z.H., Vermeulen P.B., Chaurand P., Metrakos P. (2016). Assessment of pathological response to therapy using lipid mass spectrometry imaging. Sci. Rep..

[20] Hardesty W.M., Kelley M.C., Mi D., Low R.L., Caprioli R.M. (2011). Protein signatures for survival and recurrence in metastatic melanoma. J. Proteom..

[21] Hoffmann F., Umbreit C., Kruger T., Pelzel D., Ernst G., Kniemeyer O., Guntinas-Lichius O., Berndt A., von Eggeling F. (2019). Identification of Proteomic Markers in Head and Neck Cancer Using MALDI-MS Imaging, LC-MS/MS, and Immunohistochemistry. Proteom. Clin. Appl..

[22] Kurczyk A., Gawin M., Paul P., Chmielik E., Rutkowski T., Pietrowska M., Widlak P. (2022). Prognostic Value of Molecular Intratumor Heterogeneity in Primary Oral Cancer and Its Lymph Node Metastases Assessed by Mass Spectrometry Imaging. Molecules.

[23] Boskamp T., Lachmund D., Oetjen J., Cordero Hernandez Y., Trede D., Maass P., Casadonte R., Kriegsmann J., Warth A., Dienemann H. (2017). A new classification method for MALDI imaging mass spectrometry data acquired on formalin-fixed paraffin-embedded tissue samples. Biochim. Biophys. Acta Proteins Proteom..

[24] Galli M., Zoppis I., Smith A., Magni F., Mauri G. (2016). Machine learning approaches in MALDI-MSI: Clinical applications. Expert Rev. Proteom..

[25] Kanter F., Lellmann J., Thiele H., Kalloger S., Schaeffer D.F., Wellmann A., Klein O. (2023). Classification of Pancreatic Ductal Adenocarcinoma Using MALDI Mass Spectrometry Imaging Combined with Neural Networks. Cancers.

[26] Ang K.K., Harris J., Wheeler R., Weber R., Rosenthal D.I., Nguyen-Tan P.F., Westra W.H., Chung C.H., Jordan R.C., Lu C. (2010). Human papillomavirus and survival of patients with oropharyngeal cancer. N. Engl. J. Med..

[27] Cillero-Pastor B., Heeren R.M. (2014). Matrix-assisted laser desorption ionization mass spectrometry imaging for peptide and protein analyses: A critical review of on-tissue digestion. J. Proteome Res..

[28] Wu Z., Hundsdoerfer P., Schulte J.H., Astrahantseff K., Boral S., Schmelz K., Eggert A., Klein O. (2021). Discovery of Spatial Peptide Signatures for Neuroblastoma Risk Assessment by MALDI Mass Spectrometry Imaging. Cancers.

[29] Kushner I.K., Clair G., Purvine S.O., Lee J.Y., Adkins J.N., Payne S.H. (2018). Individual Variability of Protein Expression in Human Tissues. J. Proteome Res..

[30] Rasmussen J.H., Olin A.B., Lelkaitis G., Hansen A.E., Andersen F.L., Johannesen H.H., Kjaer A., Fischer B.M., Specht L., Bentzen S.M. (2021). Intratumor heterogeneity is biomarker specific and challenges the association with heterogeneity in multimodal functional imaging in head and neck squamous cell carcinoma. Eur. J. Radiol..

[31] McGranahan N., Swanton C. (2017). Clonal Heterogeneity and Tumor Evolution: Past, Present, and the Future. Cell.

[32] Kundig P., Giesen C., Jackson H., Bodenmiller B., Papassotirolopus B., Freiberger S.N., Aquino C., Opitz L., Varga Z. (2018). Limited utility of tissue micro-arrays in detecting intra-tumoral heterogeneity in stem cell characteristics and tumor progression markers in breast cancer. J. Transl. Med..

[33] Balermpas P., Michel Y., Wagenblast J., Seitz O., Weiss C., Rodel F., Rodel C., Fokas E. (2014). Tumour-infiltrating lymphocytes predict response to definitive chemoradiotherapy in head and neck cancer. Br. J. Cancer.

[34] Eckert R.L. (1988). Sequence of the human 40-kDa keratin reveals an unusual structure with very high sequence identity to the corresponding bovine keratin. Proc. Natl. Acad. Sci. USA.

[35] Wiche G. (1998). Role of plectin in cytoskeleton organization and dynamics. J. Cell Sci..

[36] Calderwood D.A., Shattil S.J., Ginsberg M.H. (2000). Integrins and actin filaments: Reciprocal regulation of cell adhesion and signaling. J. Biol. Chem..

[37] Hailstones D.L., Gunning P.W. (1990). Characterization of human myosin light chains 1sa and 3nm: Implications for isoform evolution and function. Mol. Cell Biol..

[38] Montagna G.N., Matuschewski K., Buscaglia C.A. (2012). Small heat shock proteins in cellular adhesion and migration: Evidence from Plasmodium genetics. Cell Adhes. Migr..

[39] Gottardi C.J., Gumbiner B.M. (2001). Adhesion signaling: How beta-catenin interacts with its partners. Curr. Biol..

[40] Dowling J., Yu Q.C., Fuchs E. (1996). Beta4 integrin is required for hemidesmosome formation, cell adhesion and cell survival. J. Cell Biol..

[41] Crowe D.L., Milo G.E., Shuler C.F. (1999). Keratin 19 downregulation by oral squamous cell carcinoma lines increases invasive potential. J. Dent. Res..

[42] Lu X., Mei Y., Fan C., Chen P., Li X., Zeng Z., Li G., Xiong W., Xiang B., Yi M. (2024). Silencing AHNAK promotes nasopharyngeal carcinoma progression by upregulating the ANXA2 protein. Cell Oncol..

[43] Katada K., Tomonaga T., Satoh M., Matsushita K., Tonoike Y., Kodera Y., Hanazawa T., Nomura F., Okamoto Y. (2012). Plectin promotes migration and invasion of cancer cells and is a novel prognostic marker for head and neck squamous cell carcinoma. J. Proteom..

[44] Rong C., Muller M.F., Xiang F., Jensen A., Weichert W., Major G., Plinkert P.K., Hess J., Affolter A. (2020). Adaptive ERK signalling activation in response to therapy and in silico prognostic evaluation of EGFR-MAPK in HNSCC. Br. J. Cancer.

[45] Longuespee R., Alberts D., Baiwir D., Mazzucchelli G., Smargiasso N., De Pauw E. (2018). MALDI Imaging Combined with Laser Microdissection-Based Microproteomics for Protein Identification: Application to Intratumor Heterogeneity Studies. Methods Mol. Biol..

[46] Hartl J., Kurth F., Kappert K., Horst D., Mulleder M., Hartmann G., Ralser M. (2023). Quantitative protein biomarker panels: A path to improved clinical practice through proteomics. EMBO Mol. Med..

[47] Turck N., Vutskits L., Sanchez-Pena P., Robin X., Hainard A., Gex-Fabry M., Fouda C., Bassem H., Mueller M., Lisacek F. (2010). A multiparameter panel method for outcome prediction following aneurysmal subarachnoid hemorrhage. Intensive Care Med..

[48] Robin X., Turck N., Hainard A., Lisacek F., Sanchez J.C., Muller M. (2009). Bioinformatics for protein biomarker panel classification: What is needed to bring biomarker panels into in vitro diagnostics?. Expert Rev. Proteom..

[49] Drucker E., Krapfenbauer K. (2013). Pitfalls and limitations in translation from biomarker discovery to clinical utility in predictive and personalised medicine. EPMA J..

[50] Hemphill E., Lindsay J., Lee C., Mandoiu I.I., Nelson C.E. (2014). Feature selection and classifier performance on diverse bio- logical datasets. BMC Bioinform..

[51] Zhang Y., Bernau C., Parmigiani G., Waldron L. (2020). The impact of different sources of heterogeneity on loss of accuracy from genomic prediction models. Biostatistics.

[52] Benkarim O., Paquola C., Park B.Y., Kebets V., Hong S.J., Vos de Wael R., Zhang S., Yeo B.T.T., Eickenberg M., Ge T. (2022). Population heterogeneity in clinical cohorts affects the predictive accuracy of brain imaging. PLoS Biol..

[53] Bednarczyk K., Gawin M., Chekan M., Kurczyk A., Mrukwa G., Pietrowska M., Polanska J., Widlak P. (2019). Discrimination of normal oral mucosa from oral cancer by mass spectrometry imaging of proteins and lipids. J. Mol. Histol..

[54] Wisniewski J.R., Dus K., Mann M. (2013). Proteomic workflow for analysis of archival formalin-fixed and paraffin-embedded clinical samples to a depth of 10 000 proteins. Proteom. Clin. Appl..

[55] Tinhofer I., Stenzinger A., Eder T., Konschak R., Niehr F., Endris V., Distel L., Hautmann M.G., Mandic R., Stromberger C. (2016). Targeted next-generation sequencing identifies molecular subgroups in squamous cell carcinoma of the head and neck with distinct outcome after concurrent chemoradiation. Ann. Oncol..

[56] Hess A.K., Muer A., Mairinger F.D., Weichert W., Stenzinger A., Hummel M., Budach V., Tinhofer I. (2017). MiR-200b and miR-155 as predictive biomarkers for the efficacy of chemoradiation in locally advanced head and neck squamous cell carcinoma. Eur. J. Cancer.

[57] Bankhead P., Loughrey M.B., Fernandez J.A., Dombrowski Y., McArt D.G., Dunne P.D., McQuaid S., Gray R.T., Murray L.J., Coleman H.G. (2017). QuPath: Open source software for digital pathology image analysis. Sci. Rep..

[58] Hosmer D., Lemeshow S. (2000). Applied Logistic Regression.

[59] Sonego P., Kocsor A., Pongor S. (2008). ROC analysis: Applications to the classification of biological sequences and 3D structures. Brief. Bioinform..

[60] Zhang J., Xin L., Shan B., Chen W., Xie M., Yuen D., Zhang W., Zhang Z., Lajoie G.A., Ma B. (2012). PEAKS DB: De novo sequencing assisted database search for sensitive and accurate peptide identification. Mol. Cell Proteom..

