# Supplementary material for: Integrating MALDI-MSI-Based Spatial Proteomics and Machine Learning to Predict Chemoradiotherapy Outcomes in Head and Neck Cancer
Source: Int J Mol Sci. 2025 Sep 18;26(18):9084. doi: 10.3390/ijms26189084 (PMC12469958; doi:10.3390/ijms26189084)
Supplement: Supplementary file 1 [file ijms-26-09084-s001.zip › Supplementary Figures_Tables_Descriptions.pdf]

Supplementary Figure S1 Consort flow chart detailing patient selection for MALDI-MSI analysis.

Supplementary Figure S2 Kaplan–Meier curves showing progression-free survival (PFS) and overall survival (OS) for HNSCC patients from the ARO-0401 clinical trial included in the MALDI-MSI analysis, compared to those not included.

Supplementary Figure S3 H&E staining of exemplary HNSCC tissue samples (P-362 represents RecPro group and P-229 represents NED group). Regions corresponding to squamous cell carcinoma are marked in red.

Supplementary Figure S4 Average MALDI-MSI mass spectra $[M+H]^+$ and exemplary H&E images of investigated patient cohorts. Top: recurrence/progression (RecPro)-group; bottom: no evidence of disease (NED)-group. Tumor regions are annotated in red.

Supplementary Figure S5 Spatial distribution of predicted class probabilities for RecPro and NED groups, generated by (a) the model trained on all detected MALDI-MSI *m/z* features, and (b) the model trained on a subset of discriminatory *m/z* features identified through univariate statistical analysis (ROC). Predictions are shown across the training set for each cross-validation split. Spectra-level predictions are color-coded: red for RecPro and blue for NED, with colour intensity corresponding to prediction confidence, i.e., brighter areas indicate higher certainty. Patients were randomly assigned to test sets in each cross-validation split, ensuring that spectra from the same patient were never present in both training and test sets. Each patient appeared in the test set of only one split.

Supplementary Figure S6 Distribution and magnitude of logistic regression coefficients in the model fitted to all m/z features. (A) Coefficients fitted across each of the five cross-validation splits. (B) Mean values of the six key coefficients across the five splits. Positive mean values indicate an association of the corresponding m/z features with the NED group, while negative mean values suggest relevance to the RecPro group.

Supplementary Table S1 List of *m/z* features detected by MALDI-MSI in the CDDP-CRT tissue cohort, along with results from univariate statistical analysis. For each feature, the table provides the area under the curve (AUC) from receiver operating characteristic (ROC) analysis and the corresponding p-value from the Wilcoxon rank-sum test.

Supplementary Table S2 Tentative identification of proteins detected by MALDI-MSI showing differential intensity distributions between recurrence/progression (RecPro) and no evidence of disease (NED) groups. The full peptide library provides tentative identifications for all discriminatory *m/z* features. The short peptide library includes only proteins for which at least two corresponding peptides were identified.

Supplementary Table S3 Proteins identified by MALDI-MSI based on the consistent tissue distribution of corresponding peptides, with potential to differentiate between RecPro and NED tumor specimens in the CDDP-CRT patient cohort.

Supplementary Table S4 Logistic regression model coefficients fitted across all five splits using the complete MALDI-MSI dataset (all *m/z* features).
