# Supplementary figures and images for: Integrating MALDI-MSI-Based Spatial Proteomics and Machine Learning to Predict Chemoradiotherapy Outcomes in Head and Neck Cancer
Source: Int J Mol Sci. 2025 Sep 18;26(18):9084. doi: 10.3390/ijms26189084 (PMC12469958; doi:10.3390/ijms26189084)

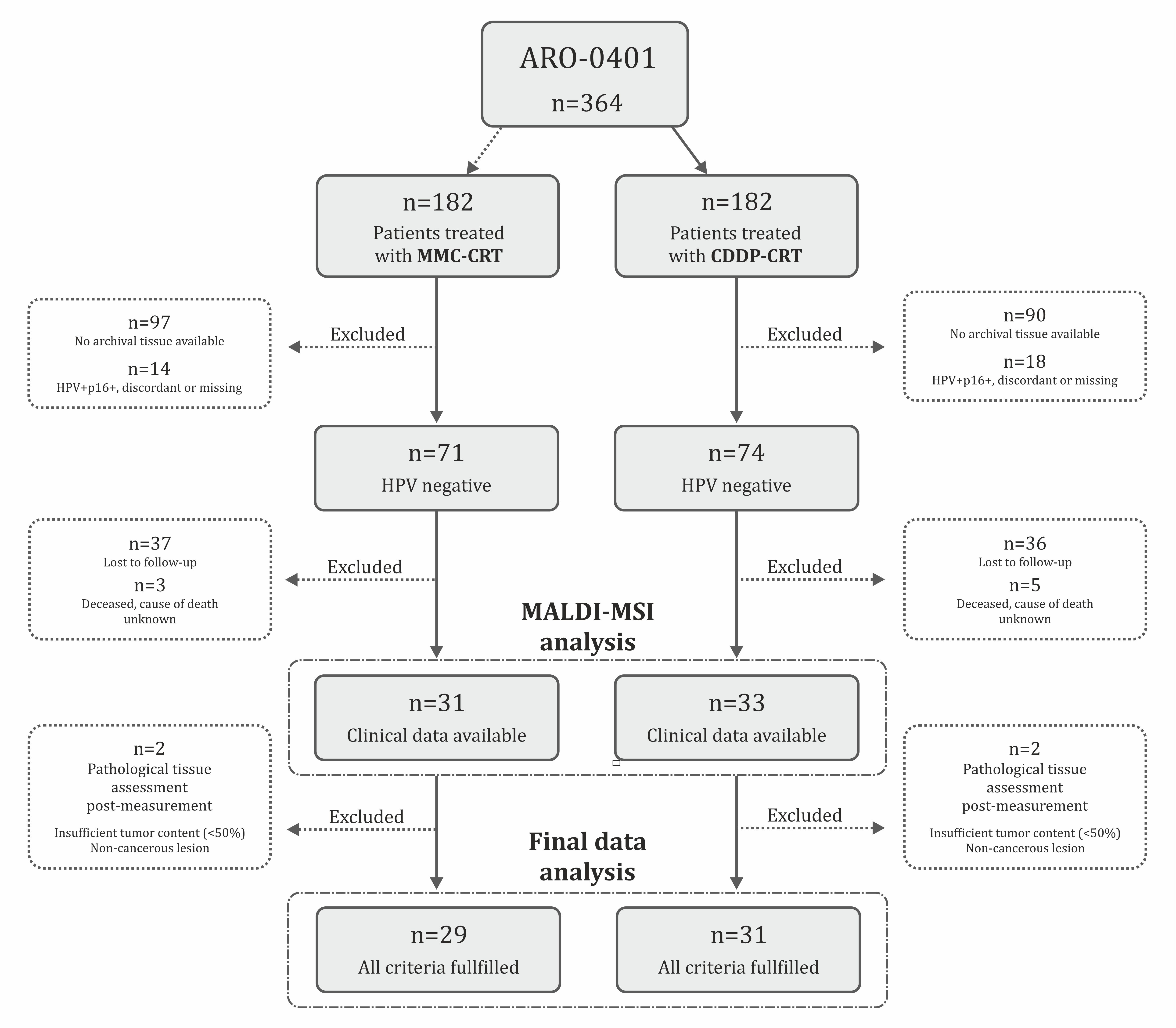

Supplement: Supplementary file 1 [file ijms-26-09084-s001.zip › Supplementary Figure S1.jpg]

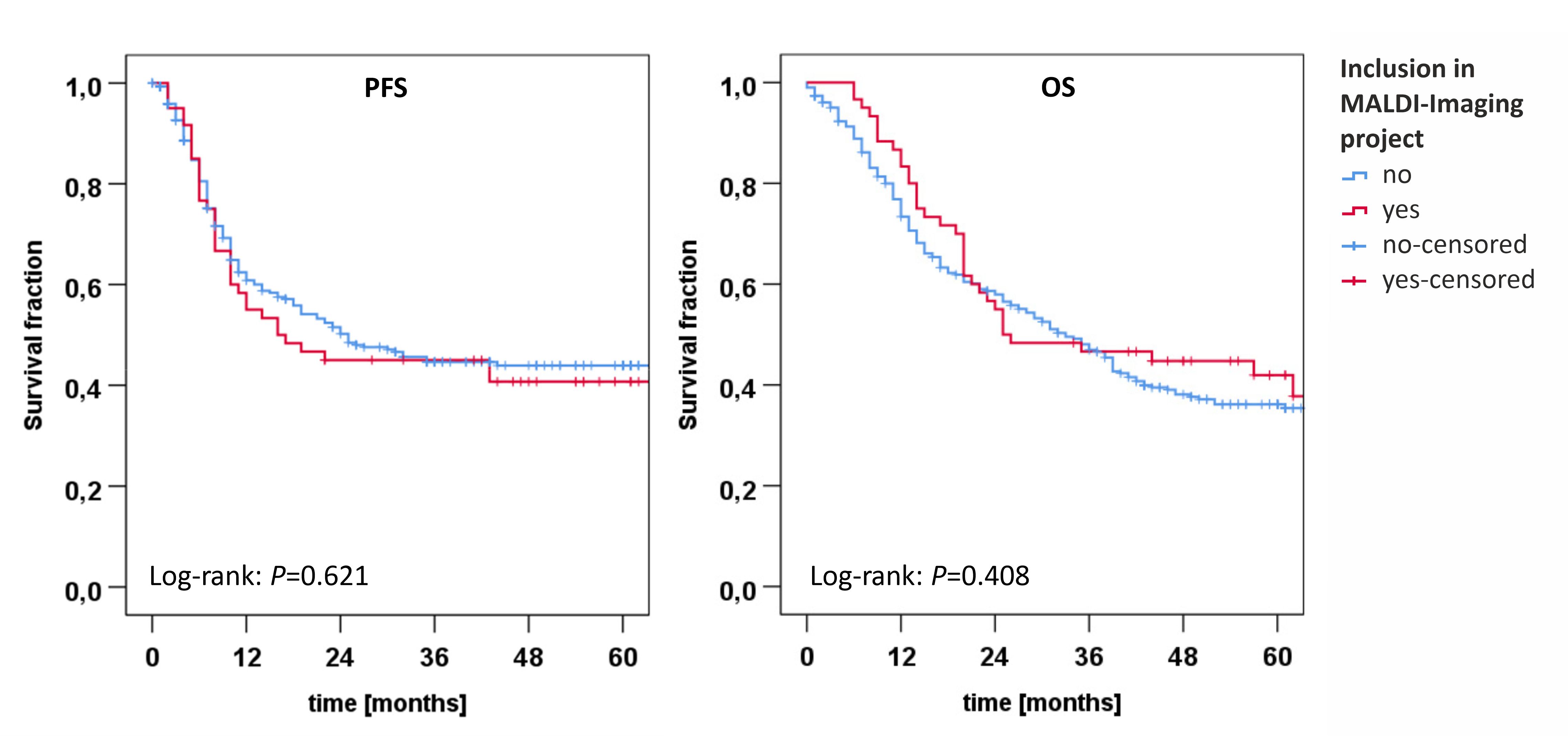

Supplement: Supplementary file 1 [file ijms-26-09084-s001.zip › Supplementary Figure S2.jpg]

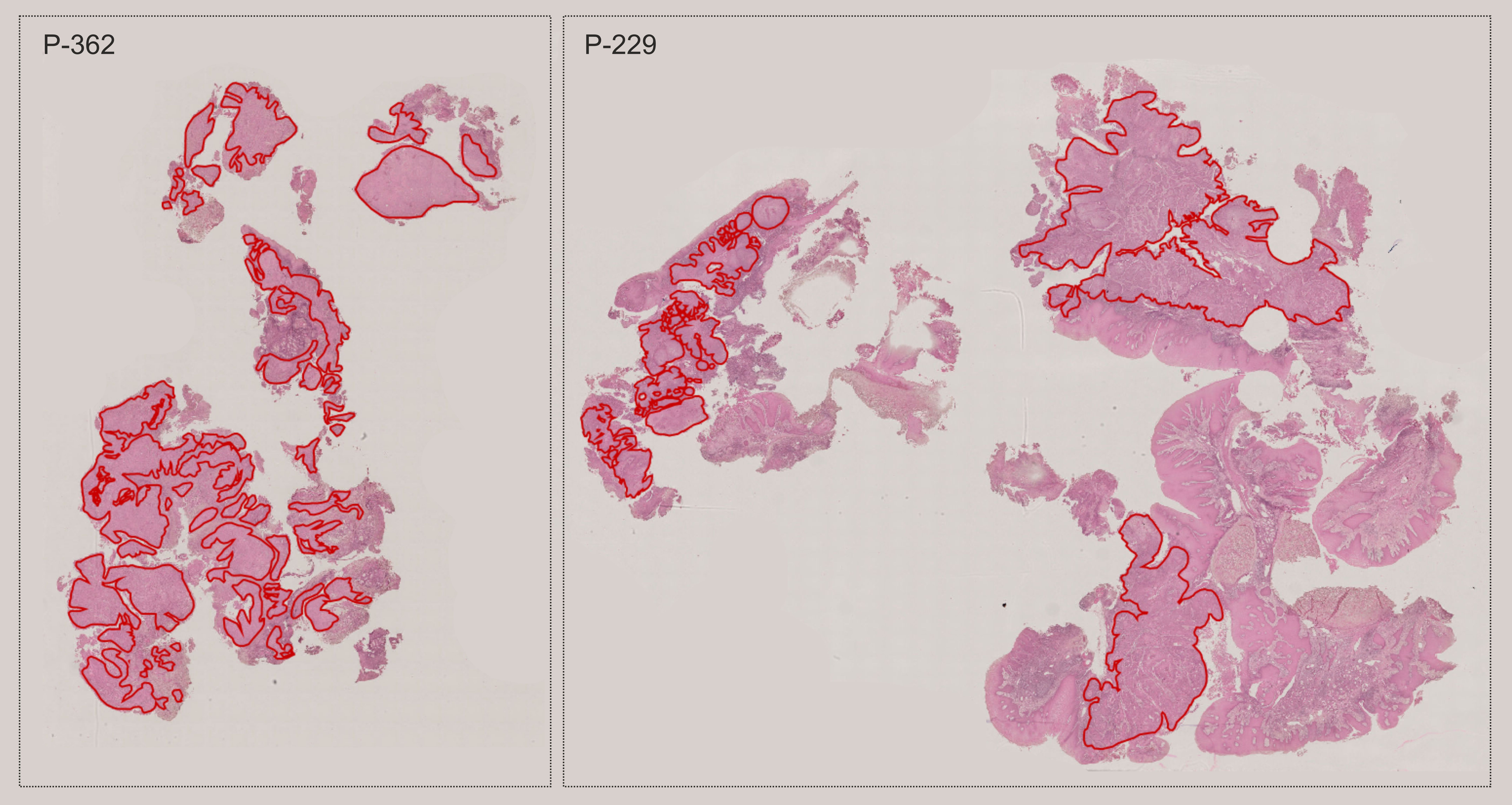

Supplement: Supplementary file 1 [file ijms-26-09084-s001.zip › Supplementary Figure S3.jpg]

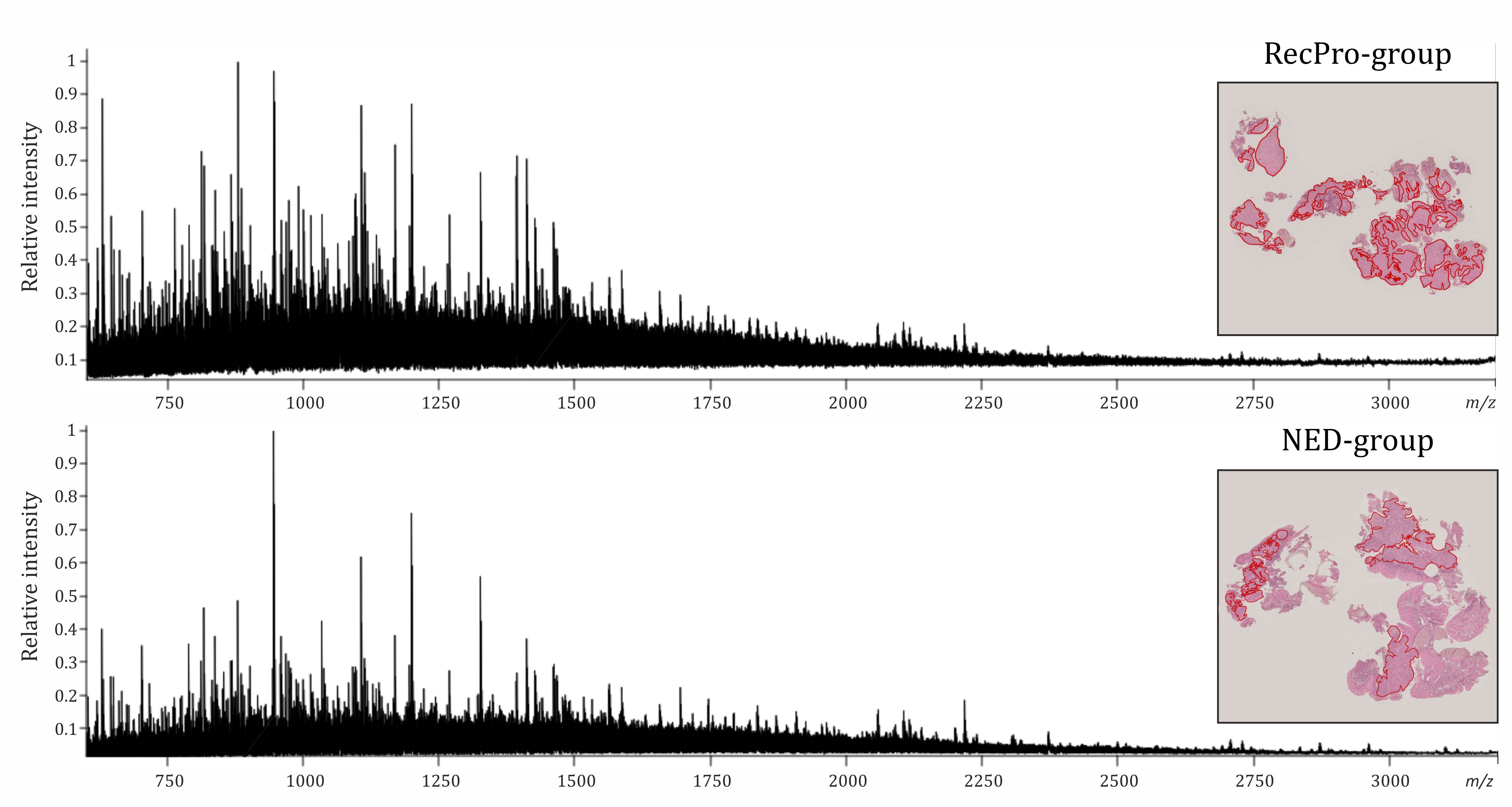

Supplement: Supplementary file 1 [file ijms-26-09084-s001.zip › Supplementary Figure S4.jpg]

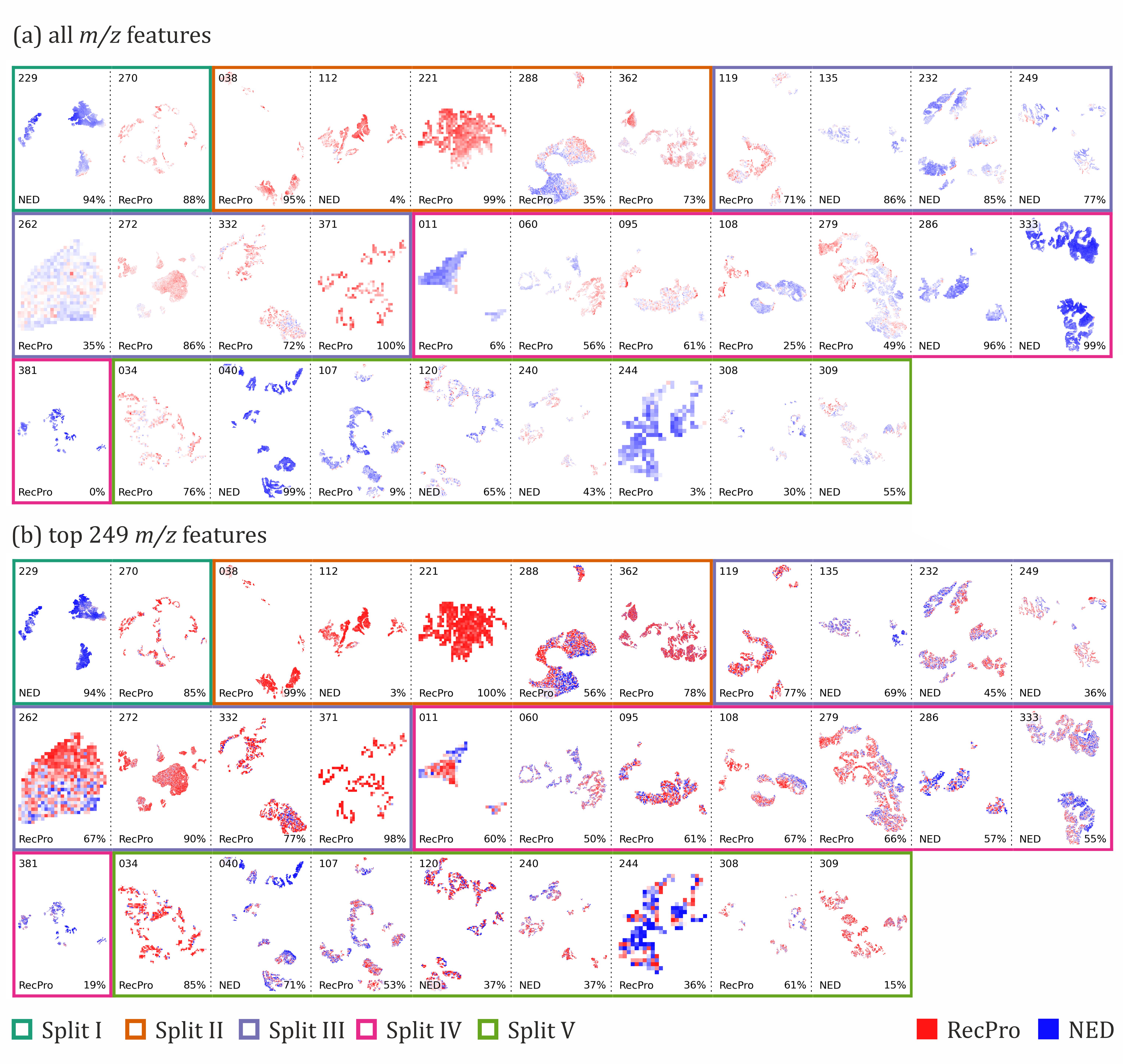

Supplement: Supplementary file 1 [file ijms-26-09084-s001.zip › Supplementary Figure S5.jpg]

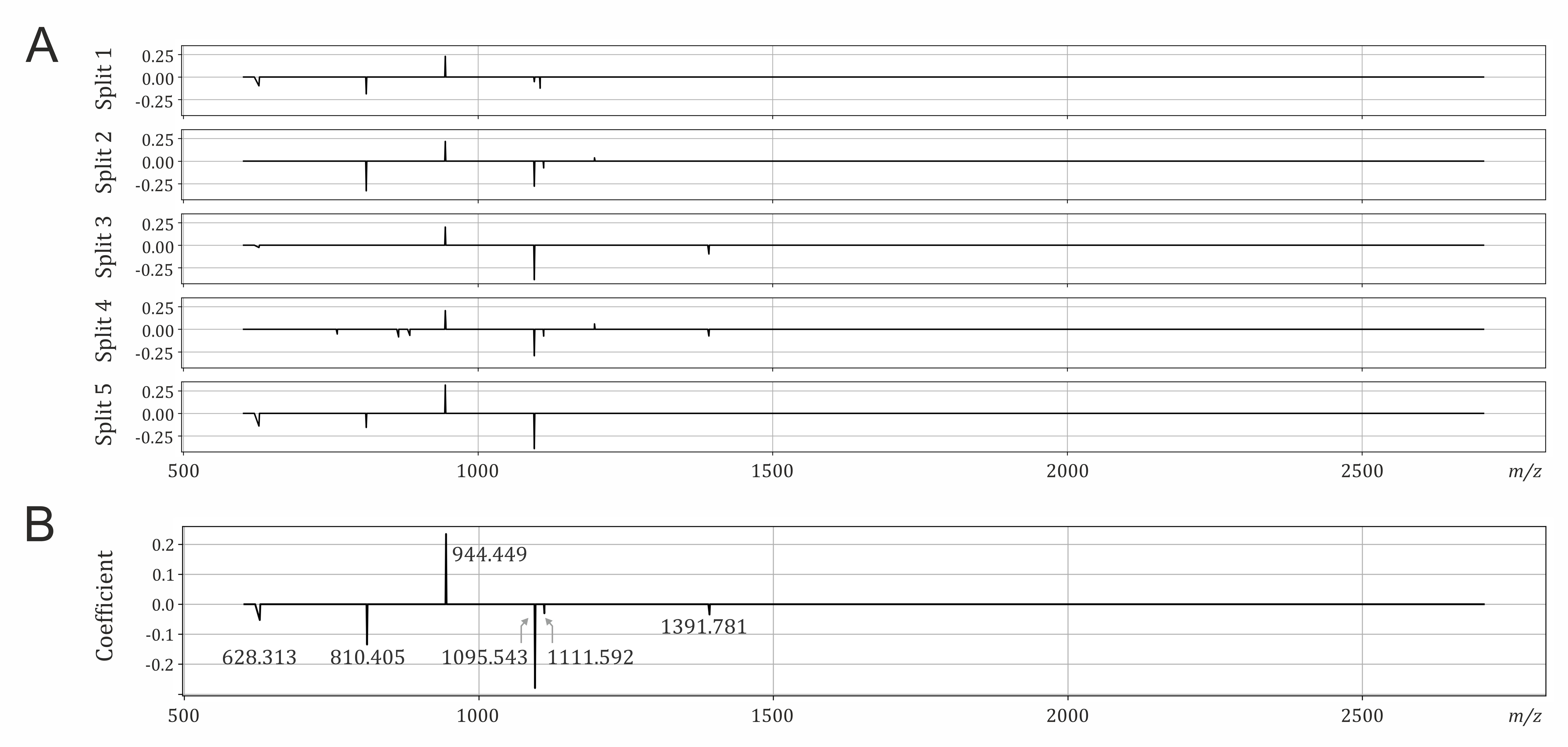

Supplement: Supplementary file 1 [file ijms-26-09084-s001.zip › Supplementary Figure S6.jpg]
